# Functional and evolutionary comparative analysis of the *DIR* gene family in *Nicotiana tabacum* L. and *Solanum tuberosum* L.

**DOI:** 10.1186/s12864-024-10577-8

**Published:** 2024-07-05

**Authors:** Tong Li, Wenbin Luo, Chaofan Du, Xiaolu Lin, Guojian Lin, Rui Chen, Huaqin He, Ruiqi Wang, Libin Lu, Xiaofang Xie

**Affiliations:** 1 College of Life Sciences, Fujian Agriculture & Forestry University, Fuzhou, 350002 China; 2 Fujian Academy of Agricultural Sciences, Fuzhou, 350003 China; 3 Longyan Tobacco Company, Longyan, 364000 China; 4 Fujian Key Laboratory of Crop Breeding by Design, Fujian Agriculture & Forestry University, Fuzhou, 350002 China

**Keywords:** Dirigent (*DIR*), Biotic stress, Expression analysis, Phylogenetic analysis, *Nicotiana tabacum* L., *Solanum tuberosum* L.

## Abstract

**Background:**

The *dirigent* (*DIR*) genes encode proteins that act as crucial regulators of plant lignin biosynthesis. In Solanaceae species, members of the *DIR* gene family are intricately related to plant growth and development, playing a key role in responding to various biotic and abiotic stresses. It will be of great application significance to analyze the *DIR* gene family and expression profile under various pathogen stresses in Solanaceae species.

**Results:**

A total of 57 tobacco *NtDIRs* and 33 potato *StDIRs* were identified based on their respective genome sequences. Phylogenetic analysis of *DIR* genes in tobacco, potato, eggplant and *Arabidopsis thaliana* revealed three distinct subgroups (DIR-a, DIR-b/d and DIR-e). Gene structure and conserved motif analysis showed that a high degree of conservation in both exon/intron organization and protein motifs among tobacco and potato *DIR* genes, especially within members of the same subfamily. Total 8 pairs of tandem duplication genes (3 pairs in tobacco, 5 pairs in potato) and 13 pairs of segmental duplication genes (6 pairs in tobacco, 7 pairs in potato) were identified based on the analysis of gene duplication events. *Cis*-regulatory elements of the DIR promoters participated in hormone response, stress responses, circadian control, endosperm expression, and meristem expression. Transcriptomic data analysis under biotic stress revealed diverse response patterns among *DIR* gene family members to pathogens, indicating their functional divergence. After 96 h post-inoculation with *Ralstonia solanacearum* L. (*Ras*), tobacco seedlings exhibited typical symptoms of tobacco bacterial wilt. The qRT-PCR analysis of 11 selected *NtDIR* genes displayed differential expression pattern in response to the bacterial pathogen *Ras* infection. Using line 392278 of potato as material, typical symptoms of potato late blight manifested on the seedling leaves under *Phytophthora infestans* infection. The qRT-PCR analysis of 5 selected *StDIR* genes showed up-regulation in response to pathogen infection. Notably, three clustered genes (*NtDIR2*, *NtDIR4*, *StDIR3*) exhibited a robust response to pathogen infection, highlighting their essential roles in disease resistance.

**Conclusion:**

The genome-wide identification, evolutionary analysis, and expression profiling of *DIR* genes in response to various pathogen infection in tobacco and potato have provided valuable insights into the roles of these genes under various stress conditions. Our results could provide a basis for further functional analysis of the *DIR* gene family under pathogen infection conditions.

**Supplementary Information:**

The online version contains supplementary material available at 10.1186/s12864-024-10577-8.

## Background

*Dirigent* genes (*DIRs*) encode proteins that act as crucial regulators of plant lignin biosynthesis and play essential roles in plant growth, development, as well as biotic and abiotic stress response [1]. The DIR protein was initially identified by Davin et al. (1997) in *Forsythia suspensa*, the DIR protein plays a crucial role in directing stereoselective bimolecular phenoxy radical coupling. This coupling reaction leads to the formation of ligna (+)-pinoresinol from *E*-coniferyl alcohol in the presence of phenol oxidase [2, 3]. DIR proteins typically possess a conserved structural domain known as dirigent, which is a distinct feature among members of the *DIR* gene family. While most *DIR* genes lack introns, a few members may occasionally contain one or two introns [4].

DIR proteins have been found to play important roles in conferring resistance to various stresses. For example, in cotton (*Gossypium hirsutum*), the overexpression of *GhDIR1* has been found to enhance resistance against the spread of *Verticillium dahlia*, a fungal pathogen associated with *Verticillium wilt* [5]. In tobacco (*Nicotiana tabacum*), the overexpression of *TaDIR13* increased the accumulation of lignin, which improved the plant resistance against *Pseudomonas clove* [6]. Moreover, the expression of *DIR* genes improved resistance against several broad-spectrum fungal pathogens, including *Leptosphaeria maculans, Rhizoctonia solani* and *Sclerotinia sclerotiorum* [7]. Additionally, *DIR* genes in sugarcane (*Saccharum officinarum*) [8], rape (*Brassica rapa*) [9], flax (*Linum usitatissmum*) [10], alfalfa (*Medicago truncatula*) [11], pear [12] have been found to play important roles in the response to various abiotic stresses, including salt, drought, low temperature, abscisic acid (ABA), jasmonic acid (JA) and other abiotic stresses. Also, DIR proteins are known to be involved in plant growth and development. For instance, a protein containing dirigent domain, has been found to be crucial for the proper formation of lignin-based Casparian strips in root [13]. Similarly, *Pdh1*, expression in soybean, plays an essential role in controlling pod dehiscence by increasing torsion in pod walls under low humidity condition. Furthermore, the overexpression of *GMDIR27* has been shown to regulate the expression of pod dehiscene-related genes, thereby promoting increased pod dehiscence [14]. Now, a comprehensive analysis of *DIR* genes has been conducted on various eukaryotic organisms on a genome-wide scale. In *Arabidopsis thaliana*, 25 *DIR* genes have been identified [3]. Similarly, in pepper (*Capsicum annuum*), 24 *DIR* genes have been reported [15]. A study on tomato (*Solanum lycopersicum*) identified 31 *DIR* genes [1], while 24 *DIR* genes were found in eggplant (*Solanum melongena* L.) [16]. Additionally, a total of 54 rice *DIR* genes were found in rice (*Oryza sativa*) [11, 17], 64 *DIR* genes in barley (*Hordeum vulgare*) [18], and 54 *DIR* genes in soybean (*Glycine max*) [14].

Solanaceae crops play a vital role in agricultural production. However, they are often threatened by various biotic and abiotic stresses. Previous studies indicated that the *DIR* genes played crucial roles in plant growth, development, and stress response [1]. Currently, there is limited study on the members of *DIR* gene family in tobacco and potato, and their exact functions remain unclear. The objectives of this study were to conduct a comprehensive investigation of the *DIR* gene family in these two crops and explore the evolutionary relationship of the plant *DIR* gene family, and thereby, revealing the expression regulation patterns of *DIR* gene family members under pathogen infection. The information obtained from this study will provide a basis for further functional analysis of the *DIR* gene family and the trait improvement of these two Solanaceae species.

## Results

### Characterization and distribution of *DIR* genes in tobacco and potato

A total of 90 *DIR* genes were identified using a BLASTP search, with 57 genes from tobacco and 33 from potato. The relevant information of these *DIR* genes and their corresponding proteins are shown in Additional file 1: Table S1, including the accession number, number of exons, protein length (aa), molecular weight (MW), isoelectric point (pI), location, and subcellular location. These tobacco and potato *DIR* family genes were renamed from *NtDIR1* to *NtDIR57* and *StDIR1* to *StDIR33*, respectively. Among the tobacco genes, the *NtDIR* proteins varied in length, ranged from 87 amino acids (*NtDIR53*) to 636 (*NtDIR10*) amino acids (aa). Correspondingly, the molecular weights varied from 9.45 kDa (*NtDIR53*) to 67.36 kDa (*NtDIR10*). The isoelectric points (pI) of the 57 NtDIR proteins ranged from 4.34 (*NtDIR53*) to 9.70 (*NtDIR18*). Among the 57 tobacco DIR proteins, 27 were classified as basic proteins (pI > 7), while 30 were classified as acidic proteins (pI < 7). In potato, the protein lengths varied greatly from 125 aa (*StDIR21*) to 401 aa (*StDIR26*). Moreover, the molecular weights ranged from 13.52 kDa (*StDIR21*) to 41.21 kDa (*StDIR26*), and the pI values ranged from 4.29 (*StDIR26*) to 9.76 (*StDIR9*). Among the 33 potato DIR proteins, 18 were identified as basic proteins (pI > 7), and 15 identified as acidic proteins (pI < 7). Furthermore, subcellular localization prediction indicated that the majority of *NtDIR* members were located in the chloroplast and extracellular regions, while the majority of *StDIR* members were located in the chloroplast.

**Fig. 1:**
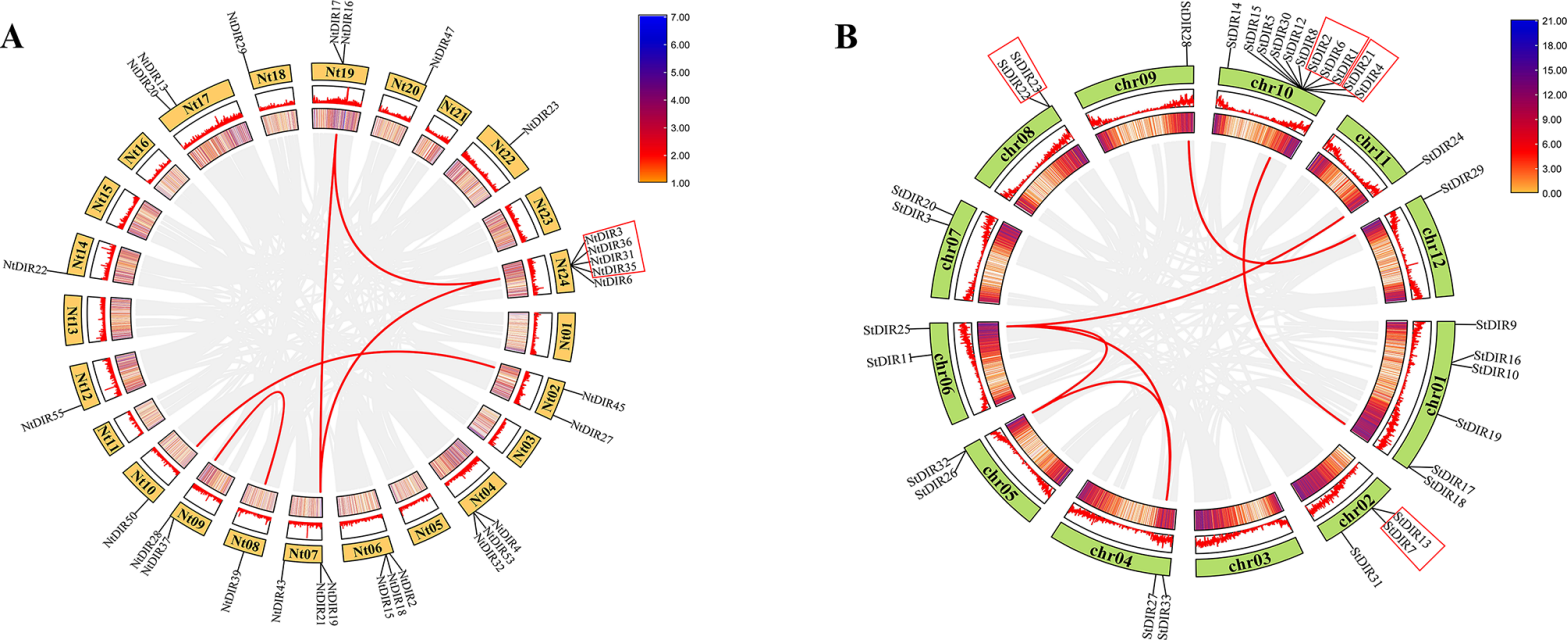
Chromosomal localization, density and syntenic relationships of *DIR* genes in two Solanaceae species. (**A**) tobacco (**B**) potato *DIR* genes are mapped on different chromosomes and syntenic gene pairs are linked by red colored lines. The red rectangles represent tandem duplication events in each species

The distribution of *DIR* genes appeared to be uneven on different chromosomes and homologous gene clusters were observed in the two investigated species (Fig. 1). In addition, the chromosome positions of some *NtDIR* genes could not be defined accurately due to the incomplete sequencing of the tobacco genome. In tobacco, a total of 29 *NtDIR* genes were unevenly distributed on 24 chromosomes of tobacco, while additional 28 genes were mapped to unattributed scaffolds (Fig. 1A). Chromosome 24 contained the biggest number of *NtDIRs* (5 genes). Similarly, a total of 33 *StDIR* genes were unevenly distributed on 12 chromosomes. Chromosome 10 had the biggest number of S*tDIR* genes (11 genes) (Fig. 1B). Furthermore, in tobacco, one cluster of homologous *DIR* genes was found on chromosome Nt24, with 4 genes within the cluster. In potato, four *DIR* gene clusters were located on chromosomes 2, 8, and 10. The number of genes within these clusters ranged from 2 to 3. The presence of multiple gene clusters may explain the expansion of the *DIR* gene family in tobacco and potato.

In addition, three pairs of tandem duplication genes on chromosome 24 (*NtDIR3/36*, *NtDIR31*/*35*, *NtDIR31*/*36*) and six pairs of segmental duplication genes (*NtDIR45*/*50*, *NtDIR19*/*17*, *NtDIR21*/*16*, *NtDIR21*/*3*, *NtDIR39*/*37*, *NtDIR16*/*3*) were identified in tobacco *DIR* gene family (Fig. 1A). In potato, five pairs of tandem duplication genes on chromosomes 2 (*StDIR7*/*13*), 8 (*StDIR22*/*23*), and 10 (*StDIR1*/*6*, *StDIR2*/*6*, *StDIR4*/*21*), along with seven pairs of segmental duplication genes (*StDIR17*/*5*, *StDIR18*/*2*, *StDIR27*/*26*, *StDIR27*/*25*, *StDIR26*/*25*, *StDIR25*/*24*, *StDIR28*/*29*) within the potato *DIR* gene family were identified in the study (Fig. 1B).

### Phylogenetics and gene structure of the *NtDIRs* and *StDIRs*

**Fig. 2:**
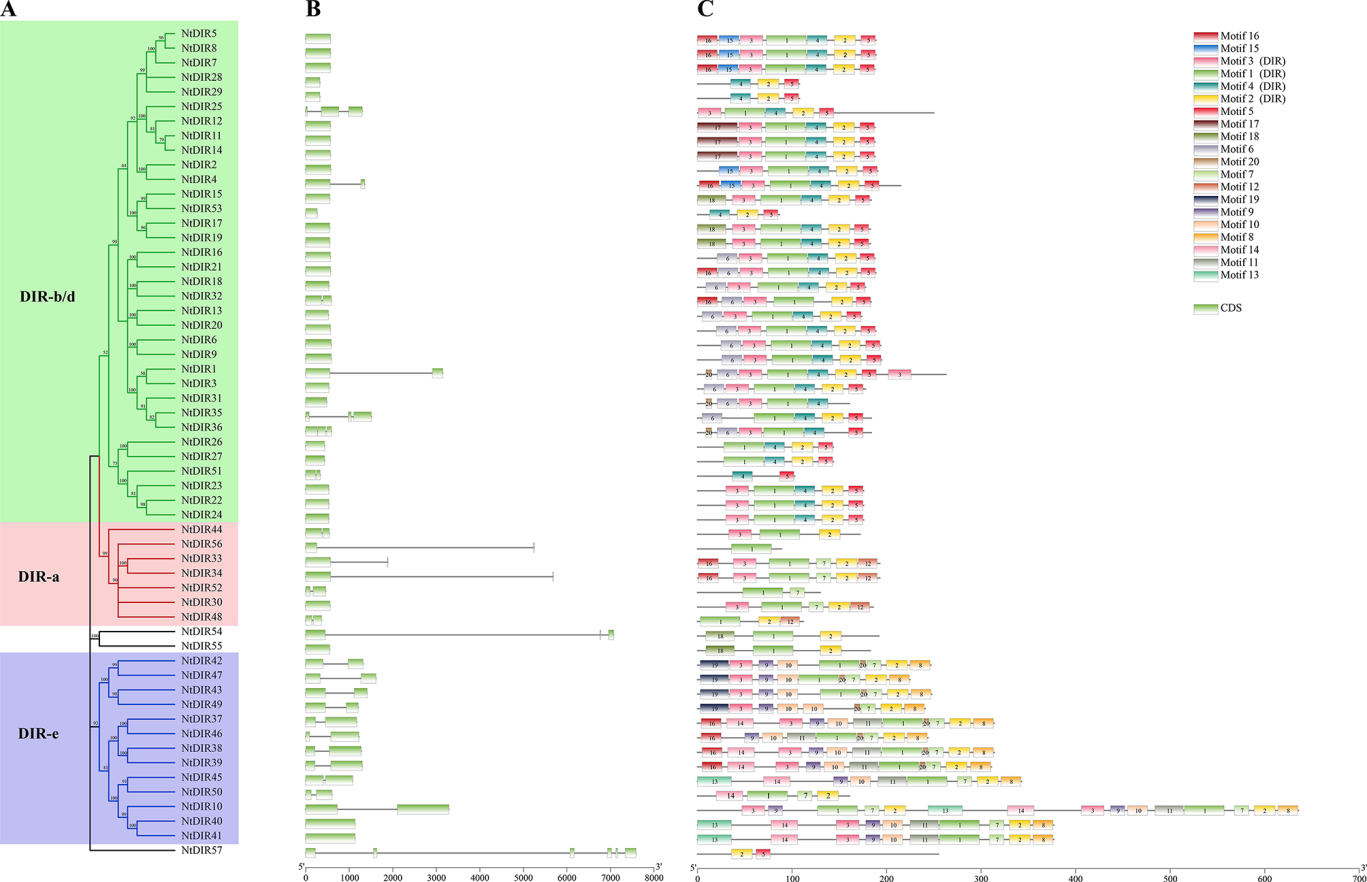
Phylogenetic tree, exon-intron structure, and conserved motifs of *NtDIRs*. (**A**) Phylogenetic tree of *NtDIRs*; (**B**) Gene structure of *NtDIRs*, exons are indicated in green, black lines represent introns; (**C**) The conserved motifs of NtDIR proteins

**Fig. 3:**
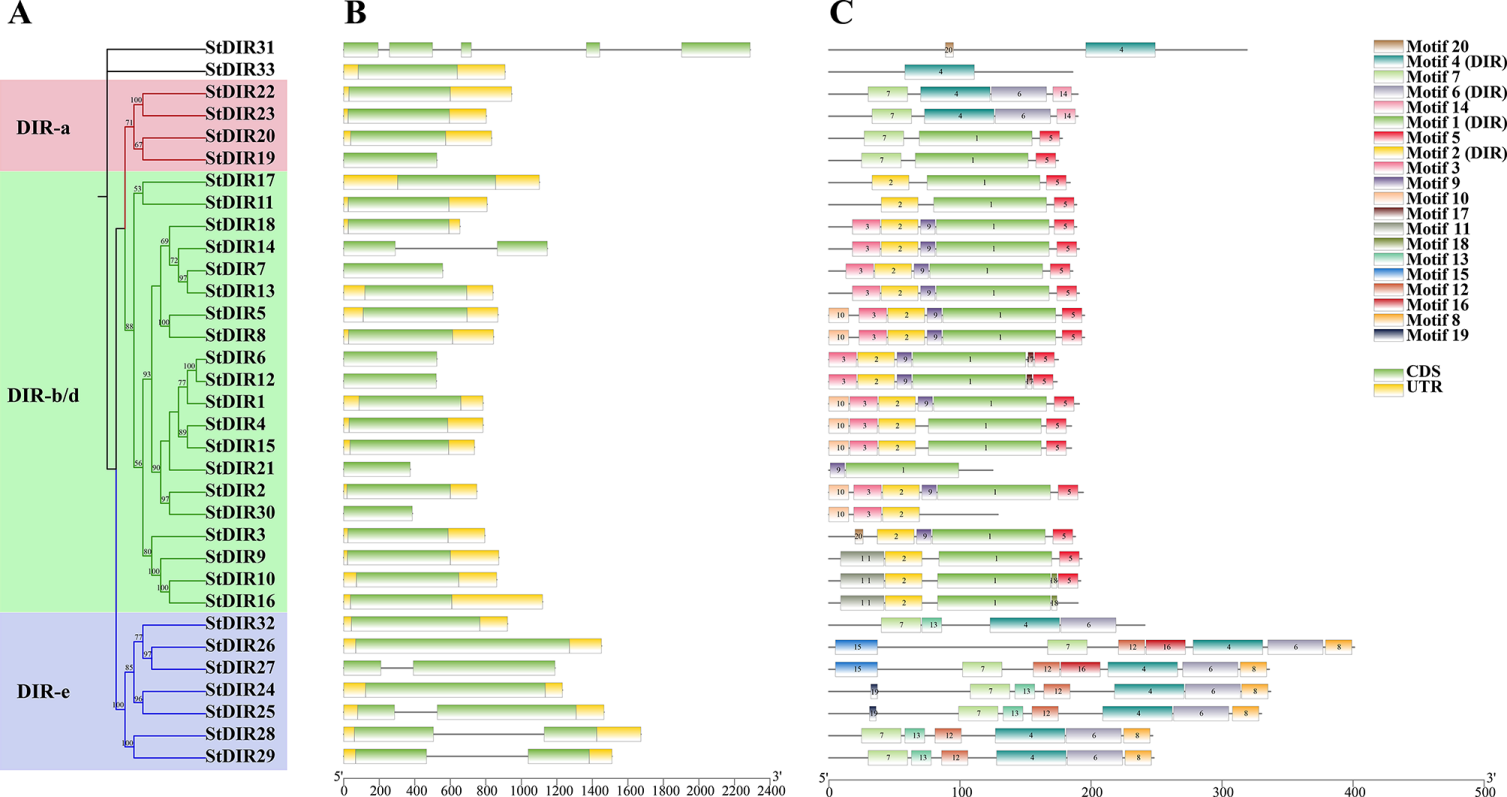
Phylogenetic tree, exon-intron structure, and conserved motifs of *StDIRs*. (**A**) Phylogenetic tree of *StDIRs*; (**B**) Gene structure of *StDIRs*. The exons are indicated in green, UTRs in yellow, and introns in black lines; (**C**) The conserved motifs of StDIR proteins

To investigate the evolutionary relationships of tobacco and potato *DIR* genes, the MEGA-X software was utilized to construct phylogenetic trees for the *DIR* gene families of both species. A total of 57 *NtDIR* genes and 33 *StDIR* genes were analyzed based on their amino acid sequences. The *NtDIRs* and *StDIRs* were categorized into three subgroups (DIR-a, DIR-b/d, DIR-e) with strong support from bootstrap (> 50%) on phylogenetic trees (Figs. 2A and 3A). However, 3 *NtDIRs* (*NtDIR54*, *NtDIR55*, *NtDIR57*) and 2 *StDIRs* (*StDIR31*, *StDIR33*) could not be assigned to any of the 3 subgroups due to low bootstrap values (< 50%). Among these subgroups, the largest group was subgroup DIR-b/d, which included 34 *NtDIRs* and 20 *StDIRs.* These *DIRs* represented more than 50% of *NtDIR* and *StDIR* members. In contrast, subfamily DIR-a, the least group, only contained 7 *NtDIRs* and 4 *StDIRs*.

Gene structure (Figs. 2B and 3B) analysis revealed variability in the number of exons among *NtDIRs* and *StDIRs.* In tobacco, *NtDIRs* exhibited exons 1, 2, 3 and 6, and *NtDIR57* exhibited the highest number of exons (6). In contrast, most *StDIR* genes in potato possessed one or two exons, with the exception of *StDIR31*, which contained 5 exons. Notably, most *DIR* genes within subgroup DIR-b/d consisted of only one exon. A significant proportion of *DIR* genes were characterized by a solitary exon (31 *NtDIRs*, 27 *StDIRs*) (Figs. 2B and 3B).

### Motif analysis of the DIR proteins in tobacco and potato

**Fig. 4:**
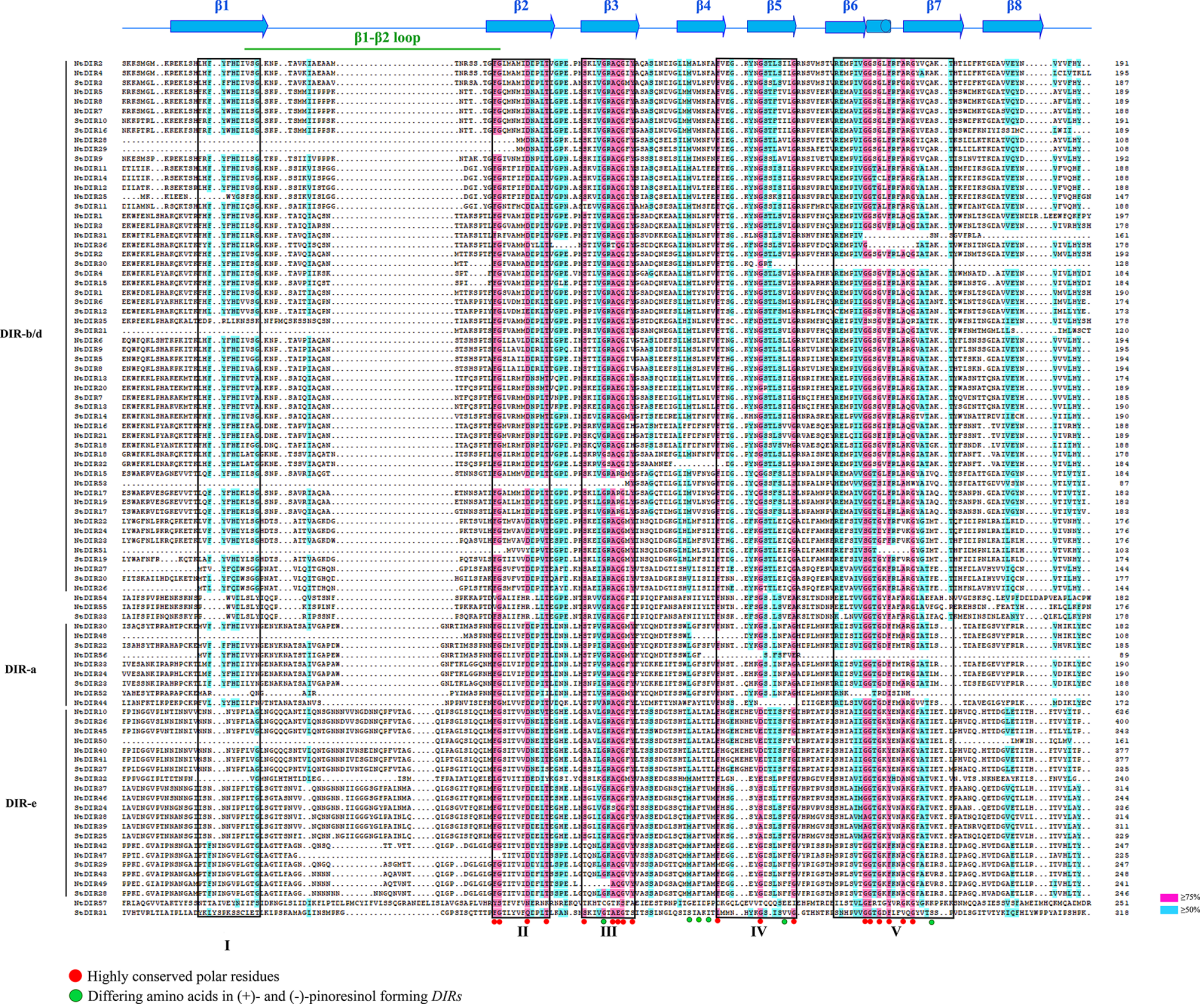
Amino acid sequence alignment of the DIR family protein sequences in tobacco and potato

Using the online MEME program, a total of 20 conserved motifs were predicted for 57 NtDIR and 33 StDIR proteins, respectively (Figs. 2C and 3C). The lengths and conserved sequences of each motif are listed in Additional file 2: Table S2. It was observed that the motif composition and distribution were relatively conservative among members within the same subgroup (Figs. 2C and 3C). For example, with the subgroup DIR-b/d, the majority of NtDIR proteins contained motifs 3, 1, 4, 2, and 5, while most of StDIR proteins contained motifs 2, 1 and 5, displaying a consistent order. In the subgroup DIR-a, motifs 1 and 7 were found in most proteins, with a consistent order, while motif 7 was present in all StDIR proteins. In the subgroup DIR-e, motifs 3, 9, 10, 1, 7, 2 and 8 were predominantly present in NtDIR proteins, following the same order. Conversely, motifs 7, 4 and 6 were identified in all of StDIR proteins in potato, displaying a uniform order within subgroup DIR-e. Notably, motifs 1, 2, 3 and 4 were associated with the DIR domain in tobacco, while motifs 1, 2, 4, and 6 corresponded to the DIR domain in potato. These results suggest that the differential distribution of conserved motifs in distinct subgroups may contribute to the evolution of functional diversity in *DIR* genes. Furthermore, the presence of similar conserved motifs among DIR proteins of tobacco and potato within the same subgroup implies potential functional similarities between these proteins. The multiple alignment among DIR protein sequences of tobacco and potato revealed the presence of five conserved motifs (I-V) (Fig. 4), originally identified in *Pinus* species by Ralph et al. [3]. These conserved sequences are located in β-strands β1 of motif I, β2 of motif II, β3 of motif III, β5 of motif IV, and β6 and β7 of the extended motif V [19, 20]. Meanwhile, several different residues between the (+)- and (−)- pinoresinol forming *DIRs* were obtained, which was in consistent with previous studies [19, 20].

### The synteny analysis of *DIR* genes in two Solanaceae species

**Fig. 5:**
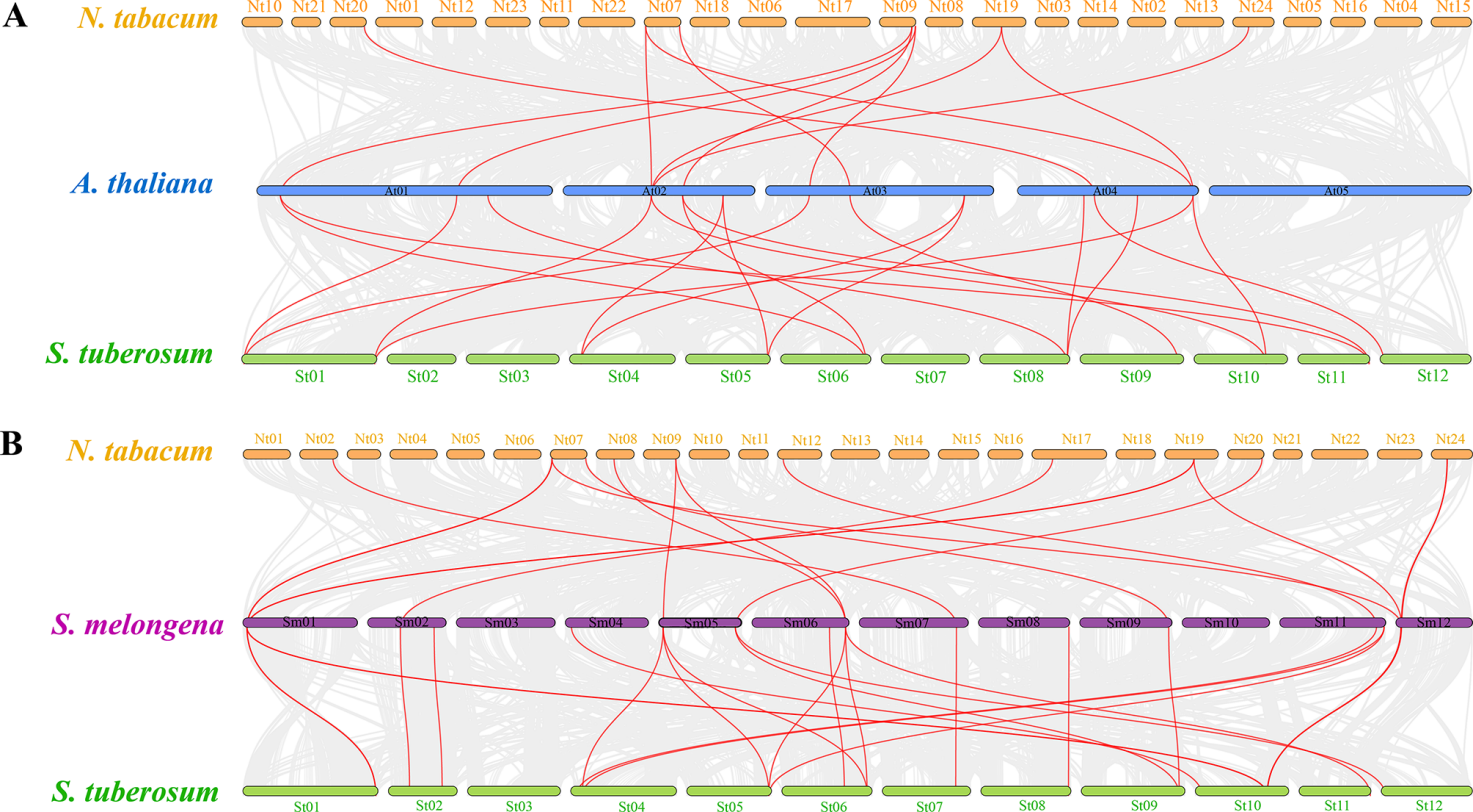
(**A**) Synteny analyses of *DIR* genes between tobacco, *Arabidopsis*, and potato. (**B**) Synteny analyses of *DIR* genes between tobacco eggplant, and potato. The gray lines in the background indicate the collinear block between each genome, while red lines highlight syntenic *DIR* gene pairs, respectively

The comparative synteny analysis of *DIR* genes across tobacco, potato, eggplant, and *Arabidopsis* was constructed by using MCScanX in TBtools software [21, 22]. A total of 11, 19, 16, and 24 orthologous gene pairs were identified in the pairs of *Arabidopsis* vs. tobacco, *Arabidopsis* vs. potato, tobacco vs. eggplant, and potato vs. eggplant, respectively (Additional file 3: Table S3). The synteny relationship was visualized by TBtools software (Fig. 5). Notably, genomic loci encompassing *NtDIR3/17/19/37/43/47* of tobacco and *StDIR2/5/17/22/24/25/26/27/28/29* of potato exhibited strong synteny conservation with their counterparts in both *Arabidopsis* and eggplant. However, 6 *NtDIRs* genes (*NtDIR13*/*16*/*21*/*27*/*39*/*55*) and 8 *StDIRs* genes (*StDIR11/13/14/15/18/20/31/33*) were associated solely with collinear gene pairs in eggplant but lacked orthologous counterparts in *Arabidopsis*. This phenomenon suggests the existence of species-specific collinear gene pairs, potentially reflecting evolutionary adaptations in Solanaceae species. Furthermore, certain *DIR* genes demonstrated associations with multiple orthologous gene pairs. For example, *StDIR26* was found to be orthologous to *AtDIR9*, *AtDIR24*, *SmDIR8*, *SmDIR13* and *SmDIR20*. Such intricate patterns of gene conservation and divergence offer valuable insights into the evolutionary dynamics and functional roles of *DIR* genes across diverse plant species.

### Comparative analysis of promoter *cis*-elements in *DIR* genes across Solanaceae species

**Fig. 6:**
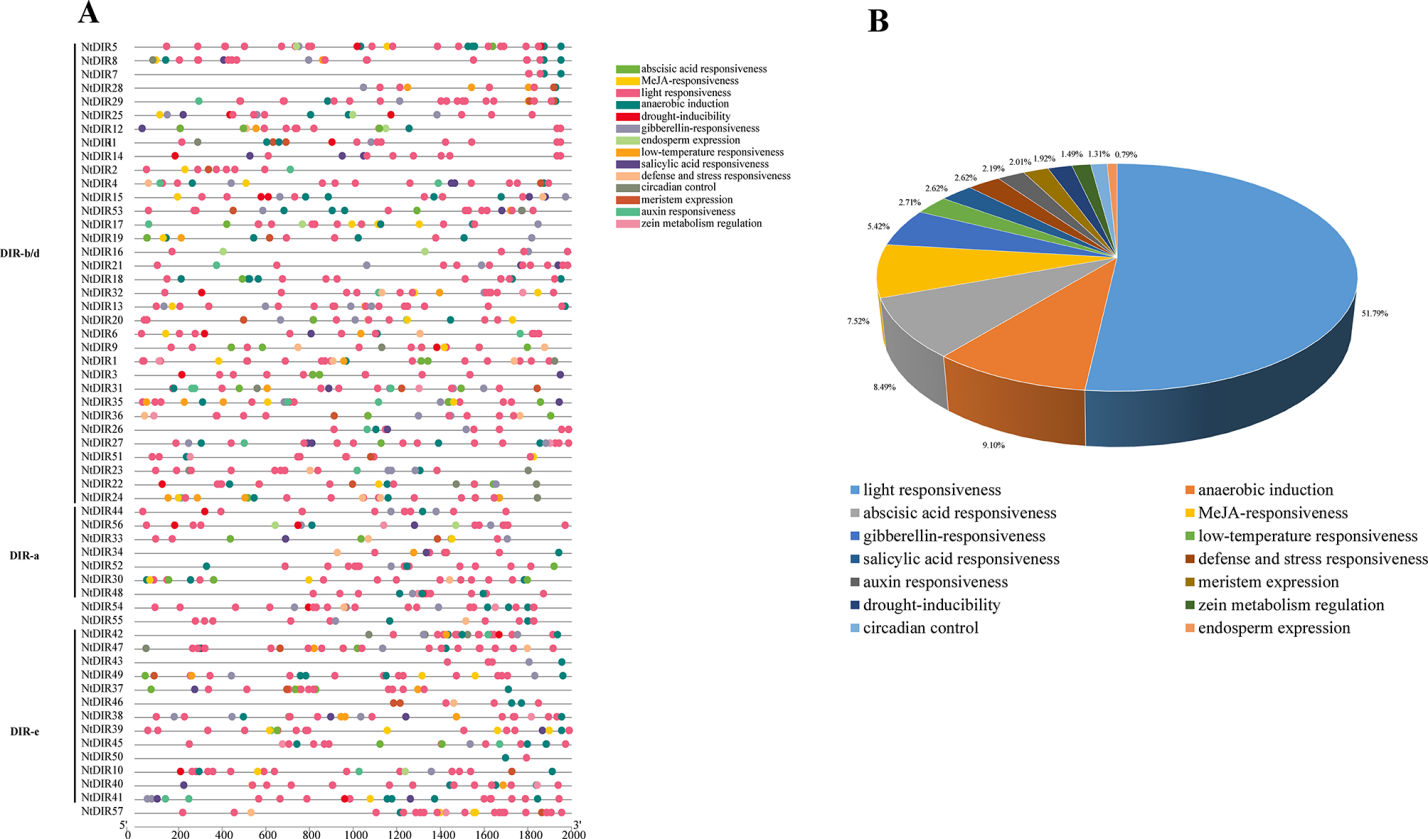
*Cis*-elements analysis of the promoters of tobacco DIR family genes. (A) The types of various *cis*-elements in the promoters of *NtDIR* genes. Different shapes and colors represent the different types of *cis*-elements. (B) The relative proportions of different kinds of *cis*-elements in the promoters of *NtDIR* genes are displayed by pie chart. Annotations of *cis*-elements were listed in Additional file 4: Table S4a

**Fig. 7:**
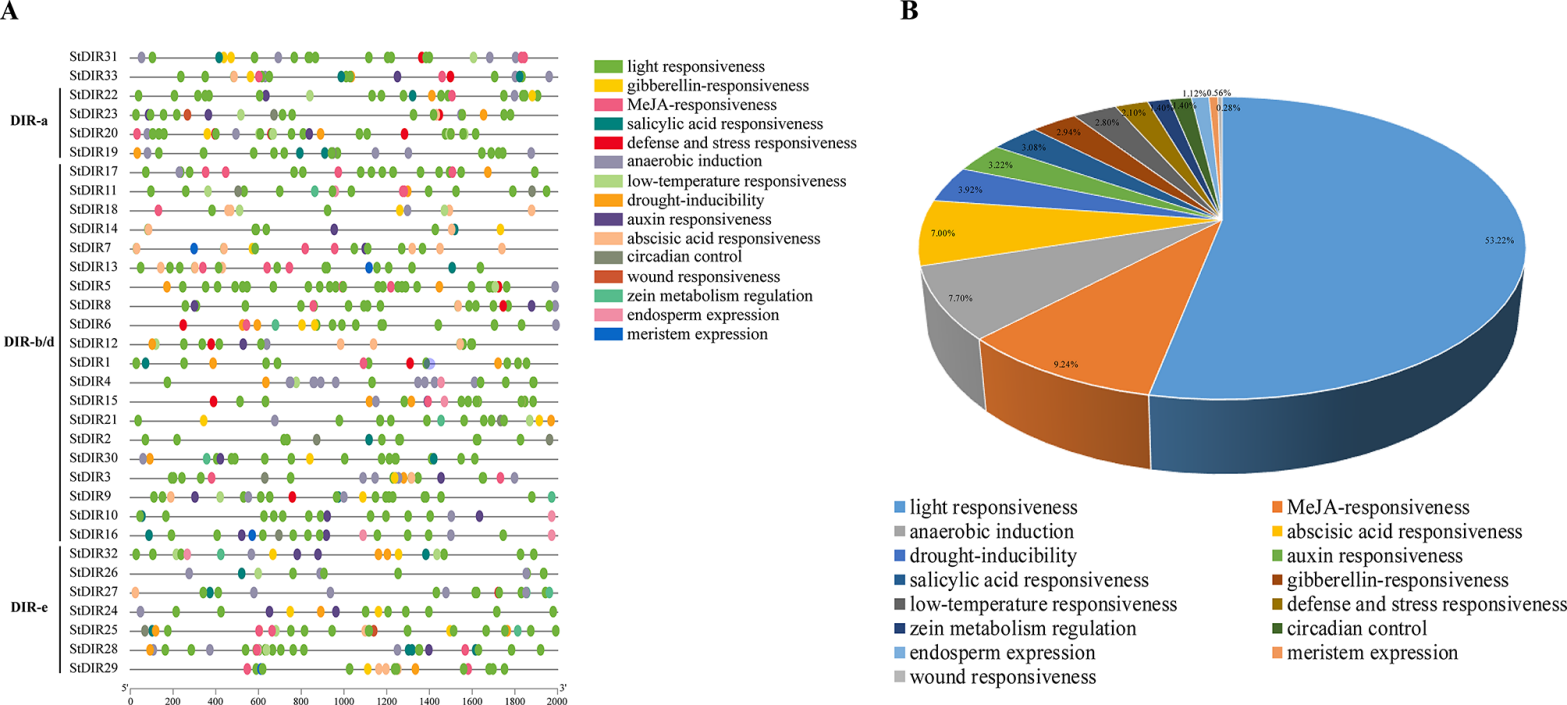
*Cis*-elements analysis of the promoters of potato DIR family genes. (**A**) The types of various *cis*-elements in the promoters of *StDIR* genes. Different shapes and colors represent the different types of *cis*-elements. (**B**) The relative proportions of different kinds of *cis*-elements in the promoters of *StDIR* genes are displayed by pie chart. Annotations of *cis*-elements were listed in Additional file 4: Table S4b

The 2 kb upstream sequences from the transcription start site of *NtDIRs* and *StDIRs* were extracted to analyze their *cis*-acting elements in the promoters. Comparative analysis revealed striking similarities in the *cis*-acting elements between these two Solanaceae species (Figs. 6 and 7). Notably, *cis*-elements associated with light responsiveness predominated in both tobacco and potato, encompassing Box 4, G-box, GT1-motif, TCT-motif, AE-box, I-box, among others, collectively constituting 51.79% and 53.22% of the total *cis*-elements for tobacco and potato, respectively. Additionally, a diverse array of *cis*-elements was identified, including those associated with hormone response (abscisic acid, MeJA, gibberellin, salicylic acid, auxin), stress responses (low temperature, defense, drought, wound), circadian control, endosperm expression, and meristem expression. A Comparison of the *cis*-elements between the two species revealed that potato possessed an additional element associated with stress response in comparison to tobacco, potentially enhancing the plant’s ability to response to biotic stress. Statistical analysis of the *cis*-acting elements in the two species showed that *NtDIR54* had the highest number of elements (33) in tobacco, whereas *NtDIR50* had the fewest (2); in potato, *StDIR25* had the highest number of elements (34), and *StDIR26* had the lowest (11). The results underscore the conserved distribution of *cis*-elements in *DIR* genes across Solanaceae species, emphasizing their indispensable roles in plant growth and development.

### Phylogenetics of the *DIR* gene family in Solanaceae species

**Fig. 8:**
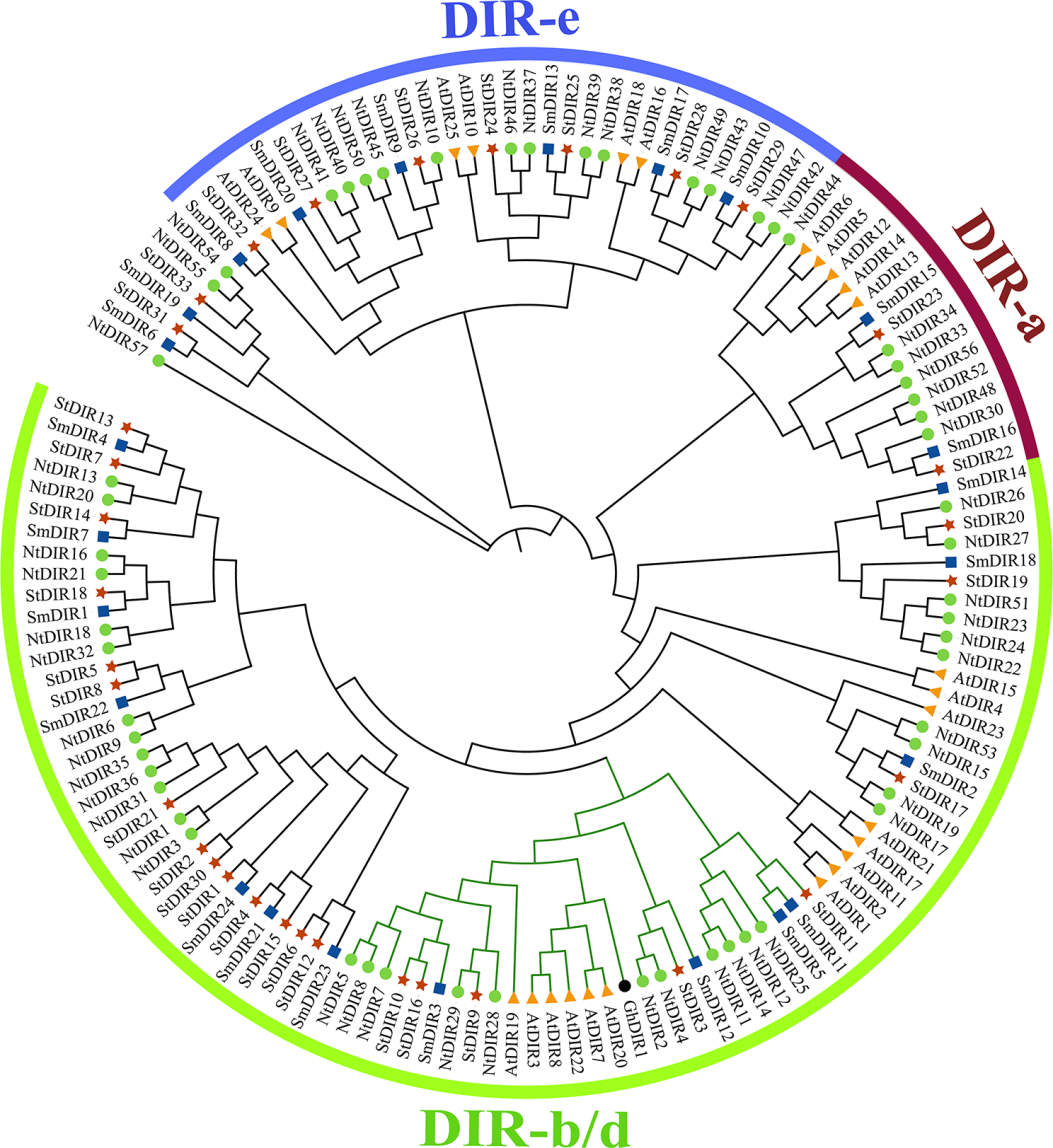
Phylogenetic tree of *DIR* genes from tobacco, potato, eggplant, *Arabidopsis*, and cotton. The nomenclature of the *DIRs* used in the tree is as follows: At, *Arabidopsis thaliana*; Nt, *Nicotiana tabacum*; St, *Solanum tuberosum*; Sm, *Solanum melongena* and Gh, *Gossypium hirsutum*. Different species are distinguished by specific color display markers. *AtDIRs* are labelled with yellow triangle markers, *NtDIRs* with green circles, *StDIRs* with red stars, *SmDIRs* with blue squares and *GhDIR1* with black circles

To elucidate the taxonomic relationships in Solanaceae species, an unrooted phylogenetic tree was constructed using 140 *DIR* gene protein sequences from diverse plant species (Fig. 8). Specifically, these sequences were obtained from tobacco, potato, eggplant [16], cotton [5] and *Arabidopsis* [3] (Additional file 5: Table S5). The phylogenetic reconstruction delineated the evolutionary clustering of the *DIR* gene family into three discernible subfamilies. Notably, the subgroup DIR-b/d was the most expansive group, comprising 14 *AtDIRs*, 34 *NtDIRs*, 22 *StDIRs* and 14 *SmDIRs* across three subgroups. Conversely, subgroup DIR-a exhibited a more constrained membership, only containing 5 *AtDIRs*, 7 *NtDIRs*, 2 *StDIRs* and 2 *SmDIRs*. Similarly, subgroup DIR-e comprised 6 *AtDIRs*, 13 *NtDIRs*, 7 *StDIRs* and 6 *SmDIRs*. In addition, the *NtDIR* and *StDIR* members were mainly distributed in the subfamily DIR-b/d, and the abundance of *DIR* members in tobacco surpassed that of *Arabidopsis* within these subfamilies, with *NtDIR* populations approximately double those observed in *Arabidopsis*. This finding corroborates the results obtained from species-specific phylogenetic analyses (Figs. 2A and 3A).

### Dynamic expression of tobacco *DIR* genes under *Phytophthora nicotianae* and *Ralstonia solanacearum* infection

**Fig. 9:**
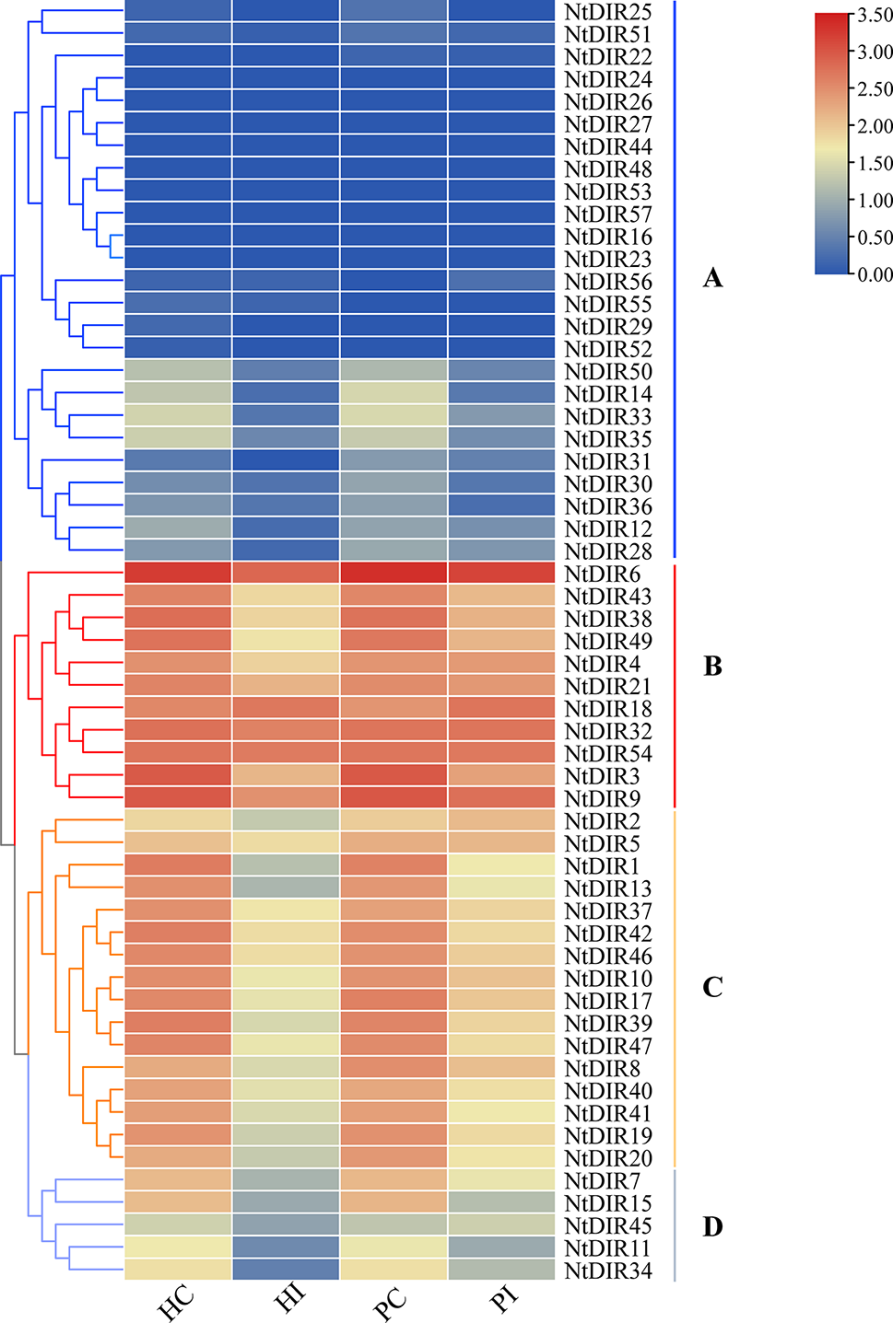
The expression of 57 *NtDIRs* in response to *Phytophthora nicotianae.* (HC: susceptible variety control; PC: resistant variety control; HI: susceptible variety at 42 h post-inoculated with *P. nicotianae*; PI: resistant variety at 42 h post-inoculated with *P. nicotianae*) FPKM values for *StDIR* genes were transformed by log_10_ (*n* + 1)

Tobacco frequently encounters disruptions due to pathogens. Utilizing the transcriptome data (GSE168854) [23], the FPKM values of *NtDIR* genes in response to the soil-borne pathogens *Phytophthora nicotianae* (*P. nicotianae*) infecting tobacco roots were obtained, and the expression profiles of 57 *NtDIR* genes were analyzed (Fig. 9, Additional file 6: Table S6). The analysis revealed diverse expression patterns among the members of *NtDIR* genes in tobacco roots under varying treatment conditions (Fig. 9). Subsequently, the expression profiles of these 57 *NtDIR* genes were categorized into four distinct clusters (A ~ D). Group A encompassed a total of 25 *NtDIR* genes exhibiting low or negligible expression levels, while group B comprised 11 *NtDIR* genes displaying high expressions. Moreover, groups C and D included 16 and 5 *NtDIR* genes, respectively. The majority of *NtDIR* genes included in groups B, C and D exhibited a down-regulated trend under the infection of *P. nicotianae* pathogen. Notably, the expression trends of *NtDIR* genes exhibited slight disparities between the susceptible (HC, HI) and resistant (PC, PI) varieties under both exposed and unexposed root conditions to *P. nicotianae* inoculum [23]. Furthermore, distinctive scenarios were identified, for instance, the expression levels of *NtDIR2* and *NtDIR45* genes displayed an increasing trend following *P. nicotianae* infection in the resistant varieties (PC, PI), while exhibiting a decrease in the susceptible varieties (HC, HI).

**Fig. 10:**
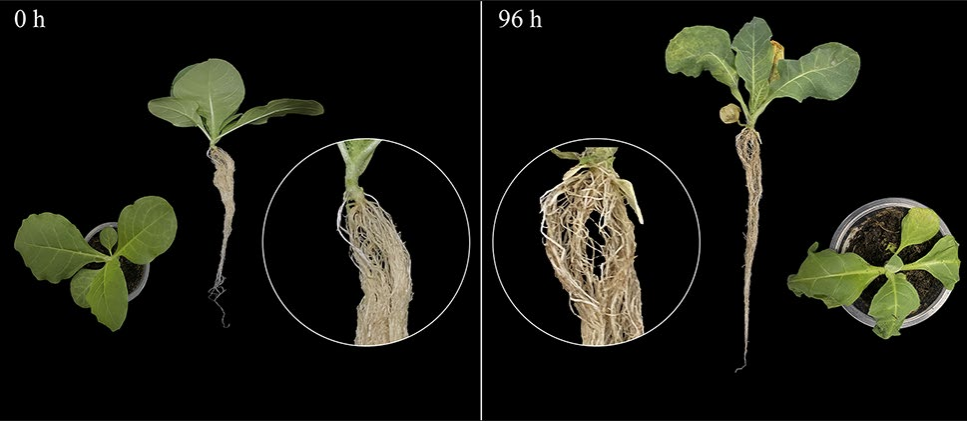
Symptoms of the cultivars Hongda 0 h and 96 h after *Ras* infection. The basal parts of the stems were magnified and shown in circles. At 0 h (left), the seedling was healthy and normal. At 96 h (right), the leaves were withered, the basal part of stem was necrotic, and the roots turned yellow

**Fig. 11:**
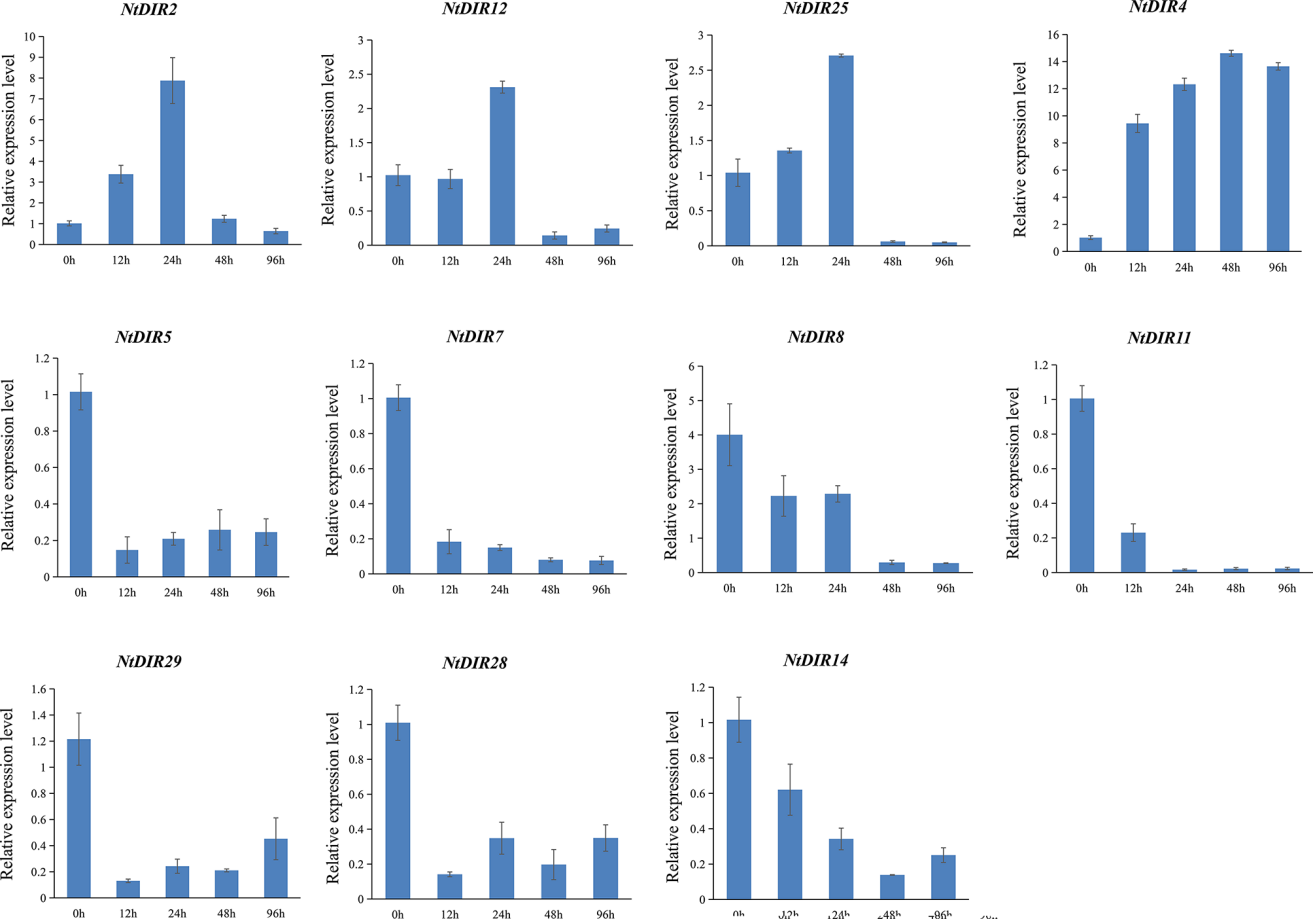
Expression profiles of *NtDIRs* in response to inoculation with *Ras*. Mean values for three replicates are shown, and the error line is the standard deviation of the three biological replicates

Moreover, tobacco bacterial wilt caused by the *Ralstonia solanacearum* (*Ras*) is one of the most serious soil-borne diseases [24]. Comparing to the 0 h, primary symptoms of *Ras* infection [25, 26], including leaf wilting, stem necrosis, and yellowing and necrosis of the roots, became evident in the seedling at 96 h post-inoculation (Fig. 10). It was reported that *GhDIR1* is mainly involved in regulating lignan biosynthesis and plays a pivotal role in pathogen resistance [5]. Consequently, it is plausible to assume that *NtDIR* genes clustered with *GhDIR1* in the same subfamily might possess similar functions. To further explore the possible function of *DIR* genes of tobacco, a total of 11 tobacco *DIR* genes that clustered with *GhDIR1* in DIR-b/d subgroups of the phylogenetic tree (Fig. 8) were selected for qRT-PCR analysis under *Ras* infection. The qRT-PCR analysis found that most of the selected genes showed significant response to *Ras* infection (Fig. 11). Most of the selected genes showed significant down-regulated expression in response to the infection (Fig. 11). Compared with the initial stage (0 h), 6 *NtDIR* genes (*NtDIR5*/*7*/*8*/*11*/*14*/*28*/*29*) were significantly downregulated at 12 h after inoculation. Specifically, the expression of *NtDIR8* and *NtDIR14* decreased gradually, compared with the sharp drop in the expression of other genes at 12 h. However, the expression of *NtDIR4* showed a notable up-regulation, exhibiting more than a 9-fold increase at 12 h post-inoculation, while the expression levels of the remaining three *NtDIR* genes (*NtDIR2*/*12*/*25*) exhibited a trend of initial increase followed by decreased with the extension of time after inoculation. The expression profiles of tobacco *DIR* genes in response to *Ras* infection unveiled functional distinctions among family members.

### Expression of *StDIR* genes in response to *Ralstonia solanacearum* and *Phytophthora infestans* infection

**Fig. 12:**
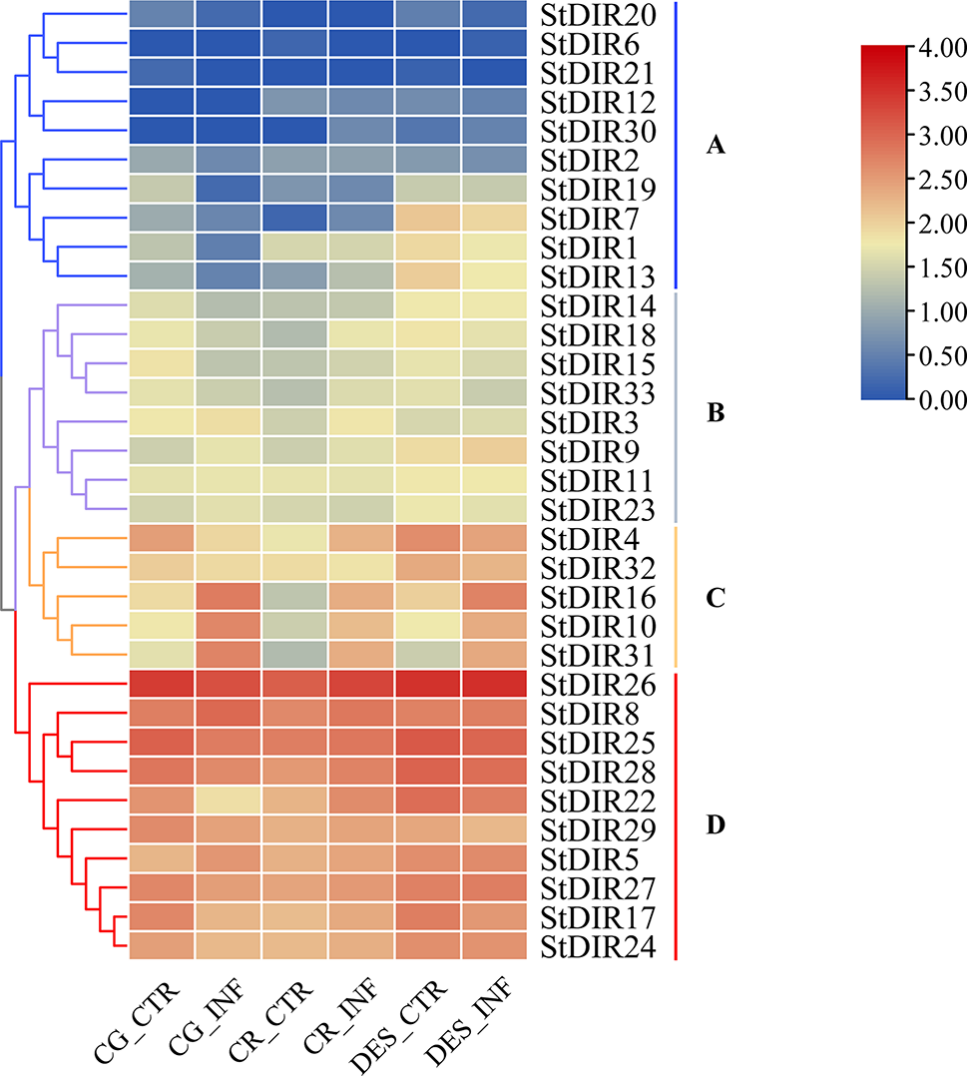
The expression of 33 *StDIRs* in response to *Ralstonia solanacearum*. (CG and CR: resistant cultivar, DES: sensitive cultivar; CTR: non-infected, INF: *Ras*-infected). FPKM values for *StDIR* genes were transformed by log_10_ (*n* + 1)

To analyze the dynamic expression profiles of *StDIR* genes in response to *Ras* infection in potato, the transcriptome data obtained from potato roots (GSE211973) under the infection of *Ras* were analyzed (Additional file 7: Table S7) [27]. The results unveiled distinct variations in the expression patterns of *DIR* genes among potato cultivars, notably the resistant CG and CR cultivars and the sensitive DES cultivar (Fig. 12). Clustering analysis revealed that 33 *StDIR* genes could be categorized into four discernible groups (A ~ D). The expression levels of ten genes within Group D were notably elevated, while Group A encompassed 10 *StDIR* genes displaying minimal or negligible expression levels. Notably, an increasing trend in the expression levels of *StDIR3*, *StDIR5*, *StDIR8*, *StDIR9*, *StDIR10*, *StDIR16*, and *StDIR31* genes was observed across all three cultivars post-*Ras* infection, contrasting with the decreased expression levels observed for *StDIR1*, *StDIR19*, and *StDIR32* genes. These findings underscore the functional diversity inherent within the *StDIR* gene family in potato, implicating their probable roles in mediating responses to *Ras* invasion, thereby contributing to our understanding of the intricate molecular mechanisms underlying potato defense strategies against *Ras*.

**Fig. 13:**
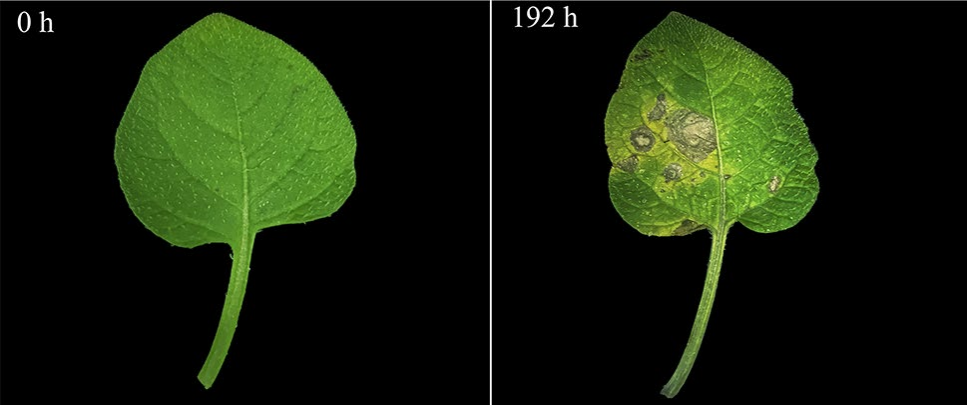
Symptoms of the line 392,278 at 0 h and 192 h after *P. infestans* inoculation

**Fig. 14:**
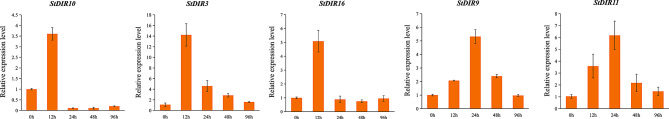
Expression profiles of *StDIRs* in response to inoculation with *P. infestans*. Mean values for three replicates are shown, and the error line is the standard deviation of the three biological replicates

In addition, potato late blight is a crucial factor affecting the yield of potato crops, which is caused by the infection of *Phytophthora infestans* (*P. infestans*). In this study, no significant changes were observed in the seedlings of potato at the initial stage of *P. infestans* infection. The main symptoms caused by *P. infestans* infection appeared in the seedling at 192 h post-inoculation (Fig. 13). During this stage, the seedlings exhibited water-stained brown spots on the leaves, which turned brown and dry and the quality of the spots was brittle and easy to crack (Fig. 13) [28]. It was reported that *GhDIR1* plays a pivotal role in pathogen resistance [5]. According to phylogenetic analysis, 5 potato *DIR* genes that clustered with *GhDIR1* in DIR-b/d subgroups of the phylogenetic tree were selected for quantitative analysis (Figs. 8 and 14). The results showed that these five selected *StDIR* genes exhibited a pattern of initially increasing and then decreasing in response to pathogen infection (Fig. 14). In particular, *StDIR10*, *StDIR3* and *StDIR16* were significantly up-regulated at 12 h post-inoculation and then down-regulated, while the response speed of *StDIR9* and *StDIR11* was slower, their highest expression levels were observed at 24 h post-inoculation. The results also highlight the diverse regulation of potato *DIR* genes in response to *P. infestans* infection.

## Discussion

*DIR* gene family members are widely distributed in various plant species. Advances in genome sequencing technology have facilitated the identification of *DIR* genes in several plant genomes, including *Arabidopsis* [3], *Capsicum annuum* [15], *Solanum lycopersicum* [1], *Solanum melongena* [16], *Oryza sativa* [11, 17], *Hordeum vulgare* [18] and *Glycine max* [14]. In this study, a total of 57 *NtDIRs* in tobacco and 33 *StDIRs* in potato were identified. Mechanisms such as polyploidization and region-specific gene duplication, including tandem repeats and segmental repeats, have been considered as important mechanisms for the expansion of plant gene families [29, 30]. This study revealed the presence of homologous gene clusters and collinear gene pairs identified in both tobacco (1 homologous gene cluster and 6 collinear gene pairs) and potato (4 homologous gene clusters and 7 collinear gene pairs), indicating that replication events were the main source of *DIR* gene family expansion in these species [29, 31]. It is worth noting that the incomplete genomic assembly of tobacco, with nearly half of the *NtDIR* genes located on scaffolds, might reduce the frequency of replication events compared to potato. Moreover, *NtDIR3* was observed in both tandem replication and collinear analysis, appearing a total of 3 times. This suggests that *NtDIR3* could potentially act as a key gene in gene replication events. In addition, the number of *NtDIR* genes in tobacco exceeds twice that in *Arabidopsis*, which may be due to the allotetraploid nature of tobacco.

Generally, the evolution of gene families is predominantly determined by the organization of gene structures, whereas within a gene family, members of the same subfamily typically exhibit high conservation in both structure and function, reflecting their evolutionary relatedness [32]. Notably, analysis of the *DIR* genes across three distinct subgroups revealed significant structural divergence, while members in the same subgroups displayed conserved gene structure and motifs [16, 33]. These observations highlight the complexity of the tobacco and potato genomes and the differentiation and diversity of the function within the *DIR* gene family. In this study, a total of 20 conserved motifs were identified in the *DIR* gene families of both tobacco and potato. Interestingly, despite belonging to the same or different subfamilies, *DIR* members exhibited variations in motif types and quantities. However, the differences within the same subfamily were notably smaller, indicating a higher level of conservation in motif composition within closely related members. Among all subgroups, motif 5 was exclusively identified in tobacco *DIRs* of the DIR-b/d subfamily, except for *NtDIR31*. Similarly, in potato, motif 2 was exclusively found in this subfamily, except for *StDIR21*. Nonetheless, all tobacco and potato members exhibited the presence of DIR motifs, indicating the conservation and diversity of *DIR* gene families across these two species. In addition, most of the tobacco (54%) and potato (82%) DIR family genes only contained one exon and lacked introns, and this result was the same with the exon/intron structures observed in *DIR* genes of other plants such as rice [17], eggplant [16] and pepper [15]. The synteny analysis of gene relationships across different species reveals that gene divergence typically precedes species divergence. Our results indicated that *Arabidopsis* had a longer divergence time from Solanaceae crops (tobacco and potato), with only 11 and 19 pairs of homologous genes identified (Fig. 5). In contrast, there were 16 and 24 homologous pairs between tobacco and eggplant, potato and eggplant, respectively. The results suggested that species with close evolutionary relationships tend to exhibit greater similarity, higher homology, and increased conservation of the *DIR* genes.

Biotic stress encompasses a range of biological factors detrimental to plant survival and development, typically stemming from infections and competition, including diseases, pests, and weeds [34]. These stress factors significantly impact crop growth and production. In the natural environment, plants must defend against attacks by pathogens, such as bacteria, fungi, viruses, nematodes, and herbivorous pests [35]. Given the severe damage caused by various diseases on crop quality and yield, mining excellent resistance genes in plants has become one of the main strategies to counter the challenge [36]. Previous studies have indicated that *DIR* gene family members play a crucial role in plant defense, making positive contributions to various disease resistance responses [15, 16, 18, 35]. In this study, a comprehensive analysis identified a total of 57 *NtDIRs* in tobacco and 33 *StDIRs* in potato and their expression was further analyzed under biotic stress. Transcriptomic data analysis revealed distinct responses among members of the plant *DIR* gene family to various pathogens, suggesting functional divergence within the family. Moreover, tobacco bacterial wilt disease was caused by *Ras*, a soilborne gram-negative bacterium [37]. Typically, the bacterium normally invades plant roots from the soil through wounds or natural openings, colonizes the intercellular space in the root cortex and vascular parenchyma, and eventually enters the xylem vessel, where they cause damage to the plant [37, 38]. Previous studies have indicated that *DIR* genes play important roles in lignans and lignin biosynthesis [1]. Hence, it is reasonable to speculate that *DIR* genes may also confer resistance to *Ras* infection. In this study, the majority of selected *NtDIR* genes showed a down-regulated expression pattern in response to *Ras* infection, except for *NtDIR2*, *NtDIR4*, *NtDIR12*, *NtDIR25*, which showed up-regulation (Fig. 11). Notably, *NtDIR4* gene exhibited strong response to *Ras* invasion, with its expression increasing more than 9 times 12 h post-inoculation, highlighting its crucial role in *Ras* disease resistance. Additionally, among these genes, *NtDIR4*, *NtDIR12* and *NtDIR25* showed down-regulation expression in response to *P. nicotianae* infection (Fig. 9, Additional file 6: Table S6). However, they exhibited significant up-regulation in response to *Ras* infection based on qRT-PCR analysis (Fig. 11), implying functional differentiation within the tobacco *DIR* gene family in response to different biotic stresses. In potato, all five selected *StDIR* genes (*StDIR3*/*9*/*10*/*11*/*16*) exhibited up-regulation in response to *P. infestans* infection (Fig. 14), and they similarly showed up-regulation in response to *Ras* infection (Fig. 12, Additional file 7: Table S7). In addition, by integrating the quantitative data from tobacco and potato, a notable observation was found: three genes (*NtDIR2*, *NtDIR4*, *StDIR3*) clustered together on subgroup DIR-b/d in the phylogenetic tree (Fig. 8) demonstrated a strong response to pathogen infection (Figs. 11 and 14). Particularly noteworthy is the inclusion of an eggplant gene (*SmDIR12*) within this cluster, which also exhibited a strong response to *Ras* infection and various abiotic stresses [16]. This result suggests that these clustered genes play an important role in responding to biotic stress. Further study of these genes would significantly contribute to an in-depth exploration of their functions in both tobacco and potato.

## Conclusion

In this study, a total of 57 *NtDIRs* and 33 *StDIRs* genes were identified in the genome of tobacco and potato, respectively, and these genes were categorized into 3 subfamilies. These *NtDIRs* and *StDIRs* were distributed randomly on 24 tobacco chromosomes and 12 potato chromosomes. A total of 3 gene pairs of tandem duplication and 6 pairs of segmental duplication were identified in tobacco based on the analysis of gene duplication events, while 5 gene pairs of tandem duplication and 7 pairs of segmental duplication in potato. *Cis*-regulatory elements of the DIR promoters participated in hormone response, stress responses, circadian control, endosperm expression, and meristem expression. Transcriptomic data analysis and qRT-PCR analysis under biotic stress revealed diverse response patterns among *DIR* gene family members to pathogen infection, indicating their functional divergence. Specifically, three clustered genes (*NtDIR2*, *NtDIR4*, *StDIR3*) exhibited a robust response to pathogen infection, highlighting their essential roles in disease resistance. This study provided valuable information for further functional exploration of *DIR* genes in tobacco and potato.

## Materials and methods

### Identification of *DIR* genes in solanaceae species

The tobacco genome data [39] was acquired from Sol Genomics Network (https://solgenomics.net/) [40]; while the potato genome data (DM v6.1) was obtained from the SpudDB website (http://solanaceae.plantbiology.msu.edu/) [41]. The 25 known *DIR* sequences from *Arabidopsis* [3], along with the HMM model (PF03018) were used as queries to retrieve candidate DIR protein sequences [15]. The tool of BLASTP (E ≤ 1e^− 10^) was used for the identification of *DIR* gene family members in Solanaceae species of tobacco and potato. The candidate protein sequences which contained conserved DIR domain (PF03018) were confirmed as the final DIR protein sequences based on the SMART (http://smart.embl-heidel-berg.de/) [42] and Web CD-search Tool (https://www.ncbi.nlm.nih.gov/Structure/bwrpsb/bwrpsb.cgi) [43]. These *DIR* genes of tobacco and potato were renamed as *NtDIRs* and *StDIRs*, respectively. The protein length (aa), protein molecular weight (MW) and isoelectric point (pI) were analyzed using Expasy ProtoParam (http://web.expasy.org/protparam/), and their subcellular locations were analyzed using WoLF PSORT (https://www.genscript.com/tools/wolf-psort) [44].

### Gene structure and conserved motif analysis

The GFF format files containing gene structures for tobacco and potato were downloaded from the Solanaceae genome database (https://solgenomics.net/) [40] and Spud DB Potato Genomics Resource (http://solanaceae.plantbiology.msu.edu/) [41]. The exon/intron structure of the *DIRs* was analyzed using TBtools software [22]. The 2 kb upstream sequences of the starting codon of *DIR* genes were submitted to the PlantCARE online program (http://bioinformatics.psb.ugent.be/webtools/plantcare/html/) for *cis*-acting elements prediction [45]. The conserved motifs of the DIR proteins were analyzed using the MEME program (https://meme-suite.org/meme/tools/meme) [46, 47], with parameters set to a motif width of 5–200 bp and a maximum number of motifs of 20 residues, while keeping the remaining parameters at default settings. Motif annotation was identified using the Pfam online tool (http://pfam-legacy.xfam.org/).

### Gene duplication and synteny analysis of DIR family genes

The potential segmental duplication and tandem duplication events were investigated using TBtools software [22]. The synteny analysis of DIR family genes among *Arabidopsis* and Solanaceae species was defined using the MCScanX method within TBtools software [21, 22]. The syntenic relationships of DIR family genes among *Arabidopsis* and Solanaceae species were graphically displayed using the TBtools software [22].

### Multiplesequence alignment and phylogenetic classification

Multiple sequence alignments of DIR amino acid sequences were performed using MEGA-X software. For phylogenetic tree construction, the amino acid sequences of DIR from different plant species were aligned using MUSCLE, and the tree was constructed using MEGA-X. The algorithm employed was the maximum likelihood method (ML), with a bootstrap value of 1000.

### Expression analysis of *NtDIR* and *StDIR* genes under various pathogens infection

To investigate the response of *NtDIR* and *StDIR* genes to pathogen infection, the transcriptome data during pathogen infection was analyzed. Using the NCBI database (https://www.ncbi.nlm.nih.gov/geo/), the FPKM (Fragments Per Kilobase of transcript per Million mapped reads) value of *NtDIR* genes under infection of *P. nicotianae* were extracted from GSE168854 [23], and the FPKM value of *StDIR* genes under the infection of *Ras* were extracted from GSE211973 [27].

Moreover, bacterial wilt is a serious disease that affects Solanaceae plants. To investigate the response of *NtDIRs* to *Ras*, tobacco seedlings at the 3–5 leaf stage were inoculated with *Ras*. Specifically, the Hongda tobacco variety was cultivated using the floating seedling method. A total of 75 tobacco seedlings were carefully selected and then inoculated with a highly virulent pathogenic strain of *Ras* that had been isolated by our laboratory [48]. Inoculation was performed by mechanically wounding the roots and followed by irrigation with 30 mL of *Ras* liquid with a concentration of 10^8^ cfu/mL [31]. These plants were then cultivated in a high-temperature and high-humidity greenhouse (30℃, about 80% humidity, 12 h/d light). Samples were collected at 0 h, 12 h, 24 h, 48 h and 96 h post-inoculation, with each biological sample comprising 5 plants and a total of 3 replicates. The seedlings were carefully uprooted, and each sample was washed with sterile water to eliminate soil and pathogens clinging to the roots. Subsequently, the surface moisture was gently blotted with a clean paper towel, the sample was wrapped in tin foil and flash-froze in liquid nitrogen, and finally stored at − 80 °C for RNA extraction. Total RNA was extracted using Hipure Plant RNA Mini Kit (Magen Biotech, Shanghai) and cDNA synthesis was performed with the SMART Kit (Takara). The expression level of *NtDIR* genes was detected by using real-time quantitative PCR (qRT-PCR) using SYBR Green qPCR premix (Universal), and the relative expression level was calculated by the 2^−∆∆t^ method [49]. The technique was repeated three times for each sample. With the tobacco actin gene as an internal reference gene, primers of *NtDIR* genes (Additional file 8: Table S8a) were designed using primer5 software (version 5.00).

Furthermore, late blight poses a significant threat to potato crops. To investigate the response of *StDIR* genes to *P. infestans* infection, potato seedlings of line 392,278 variety were propagated from in vitro cuttings, according to our previously established methods [50]. These plantlets were subsequently transplanted to the sterile substrate for cultivation. A total of 75 potato seedlings, each approximately 20 cm tall, were carefully selected and inoculated with a highly virulent pathogenic strain of *P. infestans* maintained by the Fujian Academy of Agricultural Sciences. Inoculation was performed by spraying leaves with 10 mL of sporangium liquid containing a concentration of 1 × 10^5^ sporangium/mL. The humidity level was above 95% on the initial day of inoculation and maintained around 90%, thereafter, these plants were then cultivated in a greenhouse with low temperature and low humidity (maintained at 17℃ with approximated 90% humidity, and 12 h/d light). Samples were collected at 0 h, 12 h, 24 h, 48 h and 96 h post-inoculation. Sample collection, preservation, RNA extraction, transcription and real-time quantitative analysis were consistent with tobacco. With potato actin gene as an internal reference gene, primers of *StDIR* genes were also designed using primer5 software (Additional file 8: Table S8b).

### Electronic supplementary material

Below is the link to the electronic supplementary material.

Supplementary Material 1

## Data Availability

The datasets generated and/or analyzed during the current study are available in the Gene Expression Omnibus GSE168854 (https://www.ncbi.nlm.nih.gov/geo/query/acc.cgi? acc=GSE168854) and GSE211973 (https://www.ncbi.nlm.nih.gov/geo/query/acc.cgi? acc=GSE211973).

## Abbreviations

*Ras*: *Ralstonia solanacearum* L.
*P. infestans*: *Phytophthora infestans*
*P. nicotianae*: *Phytophthora nicotianae*
MW: Molecular weight
pI: Isoelectric points
ML: maximum likelihood
*NtDIR*: *DIR* genes of *Nicotiana tabacum*
*StDIR*: *DIR* genes of *Solanum tuberosum*
FPKM: Fragments Per Kilobase of transcript sequence per Millions base pairs sequenced
qRT-PCR: Quantitative real-time PCR
