# Correction: Functional and evolutionary comparative analysis of the *DIR* gene family in *Nicotiana tabacum* L. and *Solanum tuberosum* L.

**DOI:** 10.1186/s12864-024-10628-0

**Published:** 2024-08-07

**Authors:** Tong Li, Wenbin Luo, Chaofan Du, Xiaolu Lin, Guojian Lin, Rui Chen, Huaqin He, Ruiqi Wang, Libin Lu, Xiaofang Xie

**Affiliations:** 1 College of Life Sciences, Fujian Agriculture & Forestry University, Fuzhou, 350002 China; 2 Fujian Academy of Agricultural Sciences, Fuzhou, 350003 China; 3 Longyan Tobacco Company, Longyan, 364000 China; 4 Fujian Key Laboratory of Crop Breeding by Design, Fujian Agriculture & Forestry University, Fuzhou, 350002 China

**Correction:** ***BMC Genomics*** **25, 671 (2024)**

10.1186/s12864-024-10577-8

Following publication of the original article it was reported that the name of author Libin Lu was incorrectly given as Libing Lu.

The original article has been updated.

