# Supplementary material for: Functional and evolutionary comparative analysis of the DIR gene family in Nicotiana tabacum L. and Solanum tuberosum L
Source: BMC Genomics. 2024 Jul 5;25:671. doi: 10.1186/s12864-024-10577-8 (PMC11229024; doi:10.1186/s12864-024-10577-8)
Supplement: Supplementary file 1 — Supplementary Material 1 [file 12864_2024_10577_MOESM1_ESM.zip › Files/Additional file 5/Table S5.docx]

**Table S5 The accession number of *DIRs* from other plants in our paper**

| Species | Genes | Accession number |
| --- | --- | --- |
| *Arabidopsis thaliana* L. | *AtDIR1*  *AtDIR2*  *AtDIR3*  *AtDIR4*  *AtDIR5*  *AtDIR6*  *AtDIR7*  *AtDIR8*  *AtDIR9*  *AtDIR10*  *AtDIR11*  *AtDIR12*  *AtDIR13*  *AtDIR14*  *AtDIR15*  *AtDIR16*  *AtDIR17*  *AtDIR18*  *AtDIR19*  *AtDIR20*  *AtDIR21*  *AtDIR22*  *AtDIR23*  *AtDIR24*  *AtDIR25* | At5g42510  At5g42500  At5g49040  At2g21110  At1g64160  At4g23690  At3g13650  At3g13662  At2g39430  At2g28670  At1g22900  At4g11180  At4g11190  At4g11210  At4g38700  At3g24020  CAB67637  At4g13580  At1g58170  At1g55210  At1g65870  At3g13660  At2g21100  At3g55230  At1g07730 |
| *Solanum melongena* L. | *SmDIR1*  *SmDIR2*  *SmDIR3*  *SmDIR4*  *SmDIR5*  *SmDIR6*  *SmDIR7*  *SmDIR8*  *SmDIR9*  *SmDIR10*  *SmDIR11*  *SmDIR12*  *SmDIR13*  *SmDIR14*  *SmDIR15*  *SmDIR16*  *SmDIR17*  *SmDIR18*  *SmDIR19*  *SmDIR20*  *SmDIR21*  *SmDIR22*  *SmDIR23*  *SmDIR24* | SMEL4.1_01g003540.1.01  SMEL4.1_01g003550.1.01  SMEL4.1_01g031140.1.01  SMEL4.1_02g003080.1.01  SMEL4.1_02g009260.1.01  SMEL4.1_02g018810.1.01  SMEL4.1_04g004510.1.01  SMEL4.1_05g003580.1.01  SMEL4.1_05g003590.1.01  SMEL4.1_05g019870.1.01  SMEL4.1_06g013950.1.01  SMEL4.1_06g020190.1.01  SMEL4.1_06g027190.1.01  SMEL4.1_07g016780.1.01  SMEL4.1_08g026590.1.01  SMEL4.1_08g026600.1.01  SMEL4.1_09g022870.1.01  SMEL4.1_11g000960.1.01  SMEL4.1_11g019830.1.01  SMEL4.1_11g026460.1.01  SMEL4.1_12g003420.1.01  SMEL4.1_12g003430.1.01  SMEL4.1_12g003440.1.01  SMEL4.1_12g003620.1.01 |
| *Gossypium hirsutum* L. | *GhDIR1* | ACU55135 |
