# Supplementary material for: Functional and evolutionary comparative analysis of the DIR gene family in Nicotiana tabacum L. and Solanum tuberosum L
Source: BMC Genomics. 2024 Jul 5;25:671. doi: 10.1186/s12864-024-10577-8 (PMC11229024; doi:10.1186/s12864-024-10577-8)
Supplement: Supplementary file 1 — Supplementary Material 1 [file 12864_2024_10577_MOESM1_ESM.zip › Files/Additional file 2/Table S2.doc]

| Motif | | Width | Motif sequence |
| --- | --- | --- | --- |
| 1 | 43 | | PTGFGTLTVFDDPLTEGPELNSKIVGRAQGIYGSASQDGFSLL |
| 2 | 23 | | REMPIVGGTGKFRLARGYATAKT |
| 3 | 25 | | TKLHFYFHDILSGKNPTAIAIAGAN |
| 4 | 22 | | MVLNFVFTEGKYNGSTLSILGR |
| 5 | 16 | | KTGDAIVEYNVIVLHY |
| 6 | 22 | | GVDLSPKAVEKWFKKLPHAKQK |
| 7 | 16 | | HSGEYKDSJNFFGVHR |
| 8 | 22 | | PADQHETDGVETJLHITVYLTY |
| 9 | 16 | | QVPFAKPNGFVPPVDG |
| 10 | 22 | | PNNNNNNIVNNNNVPFLTGLSG |
| 11 | 31 | | LQNNGNNNIIGGGNGQPAVTAGQLPSGJSLQ |
| 12 | 21 | | DAFEGEVYFRLRVDIKLYECW |
| 13 | 37 | | MVKFSQFCSKTFQATFCILLLALTFGIATSARILDEV |
| 14 | 29 | | SILAEEVAPPADLPTEPEPDSAPVVAPAA |
| 15 | 22 | | GEEHIFGKSIKRKSMGLKKEKL |
| 16 | 22 | | MAKLFQIKLIIILLLLLALTIT |
| 17 | 43 | | MASFRSYTLSISYFLIFSILFTPSHGAFVEEFSDILTIKRSEK |
| 18 | 31 | | MERJALLVVJLVAIFSWAEGQBKESWAKRVE |
| 19 | 31 | | QSPSSIFLTFLFLAIKFALFVSAIDTQTPGE |
| 20 | 7 | | MAFTVMF |

**Table S2** **Sequences of 20 predicted motifs of NtDIR proteins**

**Table S2** **Sequences of 20 predicted motifs of StDIR proteins**

| Motif | | Width | Motif sequence |
| --- | --- | --- | --- |
| 1 | 87 | | MIDBPLTVGPEPNSKIVGRAQGIYGSASLNEFGLLMTLNFVFTEGKYNGSTLSILGRNPVFHEYREMPIVGGSGVFRLARGYATAKT |
| 2 | 29 | | KZKVTKFHFYFHDIVSGKNPTAIQIAQAN |
| 3 | 22 | | MAQGVELGPKAVEKWFKKLPHA |
| 4 | 54 | | QQLMFGTITVFDDEJTEGHELGSSLIGKAQGFYVSSSEDGSSQTMAFTTMFHGG |
| 5 | 16 | | TTGDAIVEYNVVVLHY |
| 6 | 43 | | EYGDSLSFFGVHRIGTKESHIAVIGGTGKYKNAKGFATIKTLP |
| 7 | 31 | | LEEHTLSFYMHDILGGSNPSARAVTGIVGNP |
| 8 | 21 | | AGQHETDGVETJLHITVYLTY |
| 9 | 12 | | TSKSPTYFGLVA |
| 10 | 16 | | MEKTRLVLLLCFIVIV |
| 11 | 34 | | TISIFLSLVAFPATGEEDTYSFGKSINKKPTRLK |
| 12 | 21 | | NGANGIINNNNIPFLTGLAGT |
| 13 | 16 | | GQVPFAKPLGFVPPVD |
| 14 | 15 | | VYFRLRVDIKLYECW |
| 15 | 33 | | QFWSKTFKATFCIFLLVLSFDCANSARILDEVS |
| 16 | 31 | | QQPNTVLQNSGNNDIVNGGDNQPFVTAGQLP |
| 17 | 5 | | CWFNT |
| 18 | 5 | | HSWDF |
| 19 | 6 | | RILDEE |
| 20 | 7 | | NCHZRIW |
